# Delayed spontaneous perforation of urinary bladder with intraperitoneal seeding following radical transurethral resection of invasive urothelial cancer: a case report

**DOI:** 10.1186/1756-0500-7-167

**Published:** 2014-03-20

**Authors:** Jae Heon Kim, Won Jae Yang

**Affiliations:** 1 Department of Urology, Soonchunhyang University College of Medicine, Hospital, 59, Daesagwan-ro, Yongsan-gu, Seoul 140-743, South Korea

**Keywords:** Bladder perforation, Bladder cancer, Transurethral resection

## Abstract

**Background:**

Transurethral resection of bladder tumor (TURBT) may be applicable for the treatment of deeply invasive tumors in high-risk patients who are not suitable candidates for radical cystectomy. Intraperitoneal perforation is much less common, however, bladder wall perforation is still a cause for concerns because after perforation several studies have been reported to have peritoneal or abdominal metastases.

**Case presentation:**

An 84-year-old female patient with multiple large enhancing masses in the urinary bladder underwent radical TURBT to remove the deeply invasive tumors because she was not a suitable candidate for a major operation. Microscopic examination of the resected specimen confirmed the muscle invasive urothelial carcinoma with partial invasion into the perivesical fat tissue. She visited again 6 months later after the operation complaining abdominal distension. Computed tomography showed perforation of urinary bladder with intraperitoneal seeding and adnexa metastasis.

**Conclusion:**

Radical TURBT could be associated with delayed perforation of urinary bladder and intraperitoneal seeding.

## Background

Transurethral resection of bladder tumor (TURBT) is the primary treatment modality for bladder cancer [1]. Bladder wall perforation is considered the second most frequent complication, with a presumed incidence of 1.3% to 5% [2,3]. However, small and asymptomatic perforations of the bladder is believed to occur much more commonly than expected [4,5]. Obviously, most of these perforations are extraperitoneal, which heal spontaneously and can go unnoticed since the perforations do not cause any perioperative and postoperative problems [4,5]. Even in cases of intraperitoneal perforation, peritoneal recurrence of tumor cell has been rarely reported [3,6]. Here we describe a case of delayed perforation of the urinary bladder with intraperitoneal seeding detected at 6 months following radical TURBT.

## Case presentation

**Figure 1:**
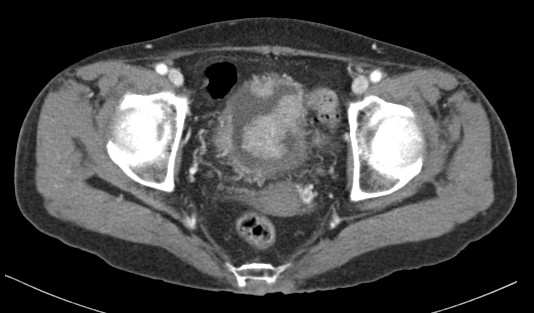
Preoperative computed tomography scan demonstrated multiple enhancing masses in the urinary bladder.

An 84-year-old female patient had been visiting our urologic clinic due to her painless intermittent gross hematuria for a year. Evaluations revealed multiple enhancing masses in the urinary bladder, measuring 3.5 × 3.0 cm and 3.3 × 3.4 cm in the left lateral wall, 2.0 × 1.3 cm in the anterior wall, and 1.2 × 1.7 cm in the right lateral wall (Figure 1). Invasion of tumor into the regional lymph node or distant organ was not seen. Under the impression of urothelial cancer of the bladder, so called “radical TURBT” was attempted to remove the deeply invasive tumors since the patient was deemed to be an unsuitable candidate for conventional radical cystectomy. Microscopic examination of the resected specimen confirmed the muscle invasive urothelial carcinoma, partly invading into the perivesical fat tissue. Cystogram conducted after the procedure revealed no extravasation of dye. The patient and her family refused major operation or any other adjuvant treatment due to her old age and comorbid physical status. The patient was urinating well and gave no specific complaint except intermittent gross hematuria during her follow up at 3 months after the operation.

**Figure 2:**
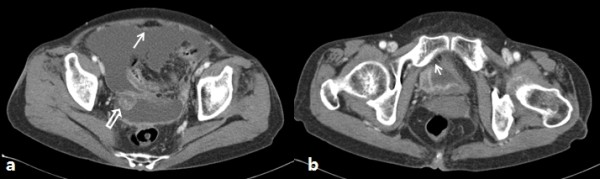
**Postoperative computed tomography. a**. Computed tomography revealed large amount of ascites with enhancing peritoneal thickening (arrow) and peripheral enhancement of the right adnexa (empty arrow). **b**. Bladder showed diffuse nodular enhancing wall thickening and focal defect in the anterior wall, suggesting perforation (arrow).

The patient visited our clinic again 6 months later after the operation complaining abdominal distension and change in her mental status. Computed tomography revealed a large amount of ascites with enhancing peritoneal thickening and peripheral nodular enhancement of the right adnexa, indicating peritoneal seeding and metastasis (Figure 2a). The urinary bladder showed enhancing wall thickening with perivesical fat infiltration and a defect in the anterior wall, suggestive of perforation (Figure 2b). Analysis of ascitic fluid demonstrated high level of creatinine (indicating urinary ascites) and was positive for metastatic carcinoma. The patient expired 7 days after admission with conservative care.

### Discussion

The objective of TURBT is to eradicate all visible disease while attaining an accurate histological diagnosis. To achieve this goal, deep and extensive resection beyond the basement membrane is generally warranted. This, however, puts the patient at risk of bladder perforation [1]. The real incidence of bladder perforation during a TURBT is not well known, and is often underestimated. This is because no routine examination is done to check bladder perforation in asymptomatic patients [7]. Two studies have described routine cystogram after TURBT [4,5]. In small series of 36 and 34 patients, contrast medium leakage was confirmed in 58.3% and 50%, respectively. All perforations were extraperitoneal and were managed conservatively [4,5]. On the other hand, intraperitoneal perforations are much less common. Bladder wall perforation is still a cause for concerns because several studies have been reported to have peritoneal or abdominal metastases after a perforation [3,6,8]. Mydlo *et al.* noted that intraperitoneal perforation was followed by extensive peritoneal and liver metastasis within 4 months of resection [3]. Bus *et al.* reported the first case of tumor seeding to both adnexa in a patient with low grade urotherlial cancer conservatively treated with TURBT that had intraperitoneal perforation [6]. Skolarikos *et al.* insisted surgical repair of a bladder perforation during transurethral resection of bladder tumor increases the risk of extravesical tumor cell recurrence and negatively affects patient prognosis, however, we assume their study had a lower level of evidence due to their retrospective approach [8]. For example, there exists a possibility that they tended to perform surgical repair to the more severe bladder perforation. All patients undergoing open surgical repair ended in extravesical tumor recurrence in their study [8].

TURBT may be applicable not only in the management of superficially invasive urothelial cancer but for the treatment of deeply invasive tumors in high-risk patients to whom the radical cystectomy is not suitable [9]. Perforation is usually a consequence of inadvertent full thickness bladder wall resection [4]. Some advocates of radical TURBT argue on the fact that since the morbidity regarding the perforation which is required for complete removal of the tumor is minimal the end justifies the means [4]. In our case, radical TURBT was attempted since the cystoscopic findings suggested invasive bladder cancer and the patient was deemed not to be a suitable candidate for a major operation. However, our patient was not a proper candidate of this procedure considering the tumor’s multiplicity, nodularity, or size [10]. Microscopic examination revealed the fat tissue that is already invaded by tumor cells, suggesting a microperforation or a very thinned-out bladder wall. We believe the latter was the case because postoperative cystogram showed no dye leakage and the patient urinated well for at least the next 3 months after TURBT. Either ingrowth of residual cancer into the bladder wall or the patient’s comorbid physical status may have prevented adequate healing of the thinned bladder wall, occurring urine leakage to be delayed. The risk of bladder perforation was associated with female gender, decreasing body mass index, higher tumor stage, deeper infiltration and higher resection weight when risk factors were separately analyzed [7]. We believe virtually all tumors invade beyond the basement membrane are high grade and allowing these cells to enter the perivesical space can jeopardize the patient [4].

## Conclusion

Radical TURBT could be associated with delayed perforation of urinary bladder and intraperitoneal seeding. We take into consideration whether this procedure is appropriate to take the risk, even though the risk of tumor implantation outside the bladder is small.

## Consent

Written informed consent was obtained from the patient’s guardians for publication of this Case report and any accompanying images. A copy of the written consent is available for review by the Editor of this journal.

## Competing interests

The authors declare that they have no competing interests.

## Authors’ contributions

JHK and WJY contributed with the conception and design of the study and drafted the manuscript. Both authors read and approved the final manuscript.
